# ﻿Molecular, chromosomal, and morphological evidence reveals a new allotetraploid fern species of *Asplenium* (Aspleniaceae) from southern Jiangxi, China

**DOI:** 10.3897/phytokeys.199.81292

**Published:** 2022-06-09

**Authors:** Chen-Xue Lin, Guo-Liang Xu, Zhi-Fang Jin, Wen-Bo Liao, Ke-Wang Xu

**Affiliations:** 1 Co-Innovation Center for Sustainable Forestry in Southern China, College of Biology and the Environment, Nanjing Forestry University, Nanjing, 510275, China; 2 Jiulianshan National Nature Reserve Administrative Bureau, Longnan, 341700, China; 3 State Key Laboratory of Biocontrol, School of Life Sciences, Sun Yat-sen University, Guangzhou, 510275, China; 4 Guangdong Provincial Key Laboratory of Plant Resources, School of Life Sciences, Sun Yat-sen University, Guangzhou, 510275, China

**Keywords:** Black-stemmed spleenworts, conservation, new taxon, species complex, taxonomy

## Abstract

*Aspleniumjiulianshanense*, a new tetraploid fern species of the *A.normale* complex (Aspleniaceae) from Jiulianshan National Nature Reserve, southern Jiangxi, China is described and illustrated. We inferred the phylogenetic position of the new species based on sequences from seven plastid markers (*atpB*, *rbcL*, *rps4*, *rps4-trnS*, *trnL*, *trnL-F*, and *trnG*) and one low-copy nuclear gene, *pgiC*. The plastid phylogeny supported a close relationship among the new species *A.jiulianshanense*, *A.minutifolium*, and *A.kiangsuense*, while the nuclear phylogeny differed in topology from the plastid tree. The new species may be due to hybridization between *A.kiangsuense* and *A.boreale*. Morphologically, the new species can easily be distinguished from other members of the *A.normale* complex by rachises bearing a gemma near the apex, pinna margins entire to sparsely crenate, and (1‒)3‒4(‒6) sori per pinna.

## ﻿Introduction

*Asplenium* L. (Aspleniaceae) is one of the largest fern genera, comprising more than 700 species (Xu et al. 2020). Hybridization and polyploidization events tend to occur in the genus and hence generate a great number of species complexes (Schneider et al. 2017; Chang et al. 2018). *Aspleniumnormale* D. Don and its affinities constitute a taxonomically challenging species complex in the genus (Chang et al. 2013, 2018). Species delimitation in the *A.normale* complex was poorly understood until an integrative taxonomic approach using cytological, morphological, and DNA sequence data was employed to delimit species in the complex (Chang et al. 2013, 2018; Fujiwara et al. 2017, 2020; Chang et al. 2020).

Members of the *A.normale* complex are widely distributed in south and southeast Asia, tropical east Africa, and tropical Pacific islands (Lin and Viane 2013). Previous floristic treatments tended to use a broadly defined species circumscription including a single widespread species *A.normale* (Lin and Viane 2013) and a few narrowly distributed species such as the Chinese endemic *A.kiangsuense* Ching & Y.X.Jin (Lin and Viane 2013), the Japanese endemic *A.oligophlebium* Bak. (Matsumoto et al. 2003), and the Hawaiian endemic *A.hobdyi* W.H.Wagner (Ranker et al. 2019). However, recent studies revealed that the broadly defined species circumscription overlooked some cryptic species in the complex and was inappropriate (Chang et al. 2013, 2018; Fujiwara et al. 2017). Some cryptic species and subspecies have been recognized and described consequently based on morphological, cytological, and molecular evidence in recent studies (Li et al. 2016; Chang et al. 2018, 2020; Fujiwara et al. 2020).

**Figure 1.**
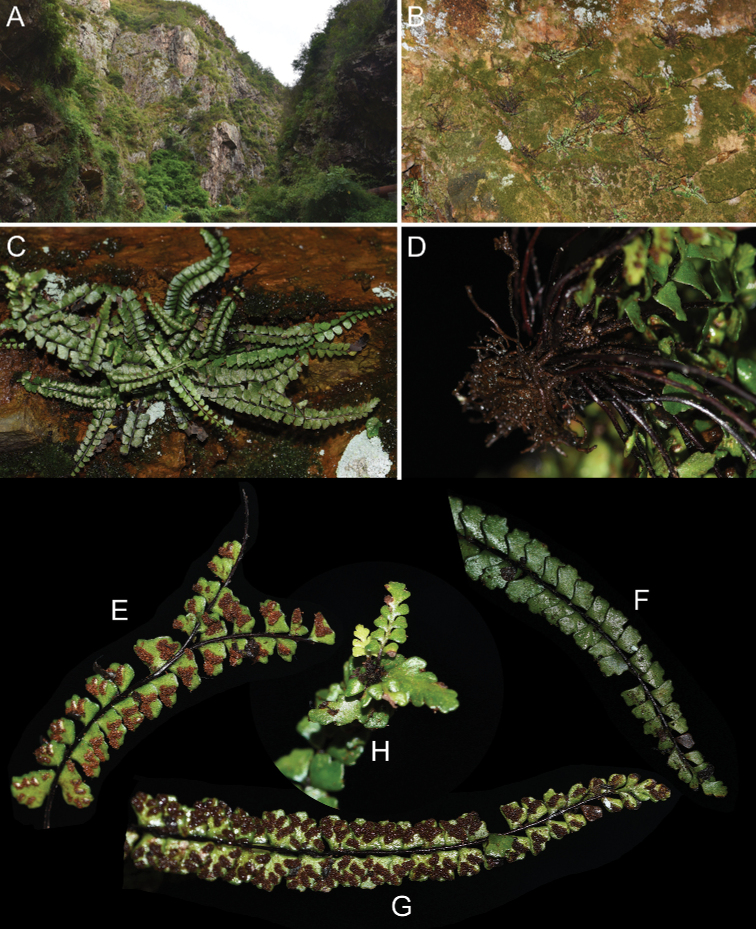
*Aspleniumjiulianshanense* K.W.Xu & G.L.Xu **A, B** habitat where the new species was discovered **C** habit **D** rhizome and roots **E** irregular branch of lamina **F** adaxial view of portion of lamina **G** abaxial view of portion of fertile lamina **H** frond with a gemma at the distal end of the rachis.

**Figure 2.**
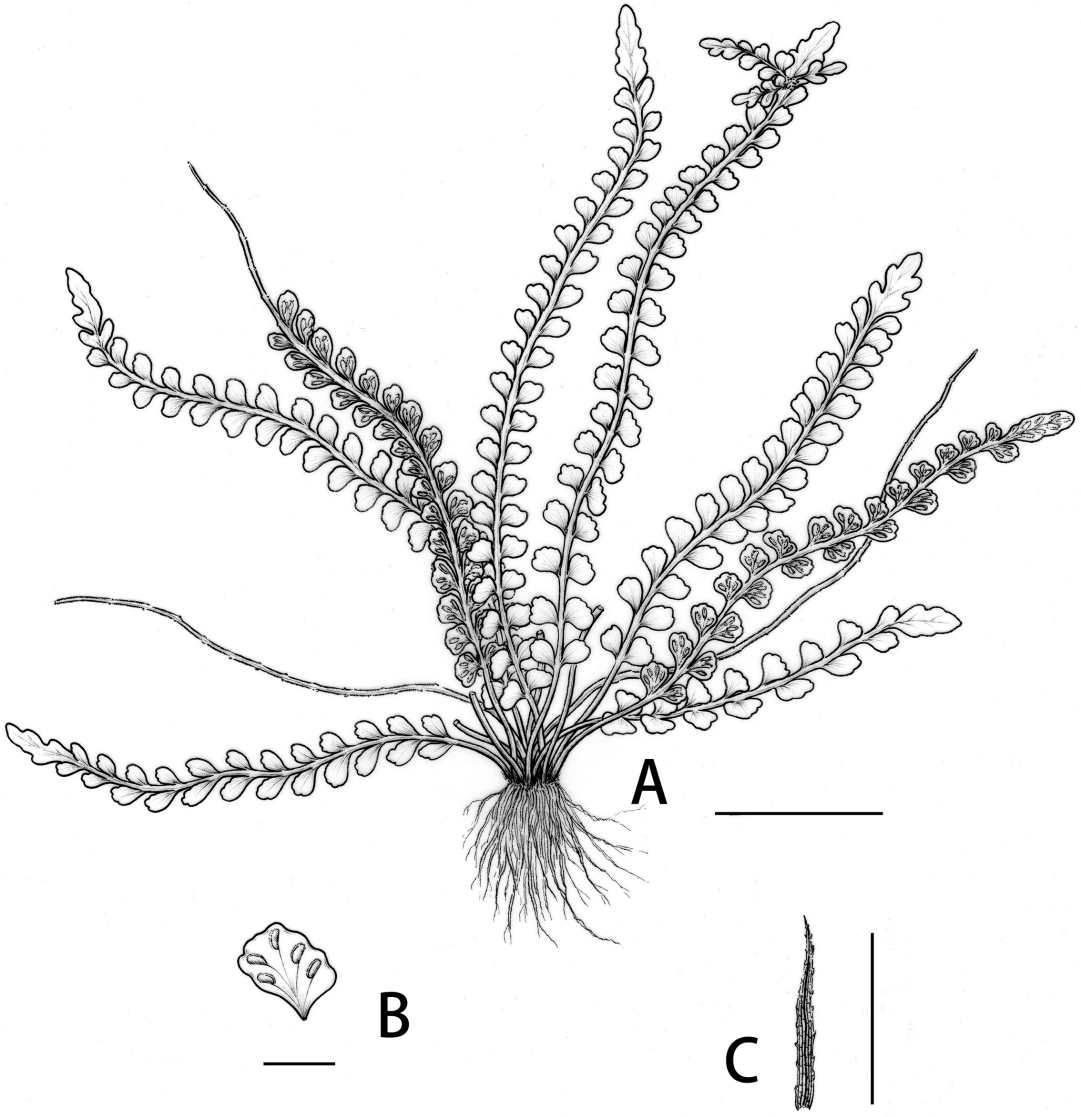
*Aspleniumjiulianshanense* K.W.Xu & G.L.Xu **A** habit **B** pinna showing the venation and the distribution of sori **C** rhizome scale. Scale bars: 2 cm (**A**); 5 mm (**B**); 2 mm (**C**).

**Figure 3.**
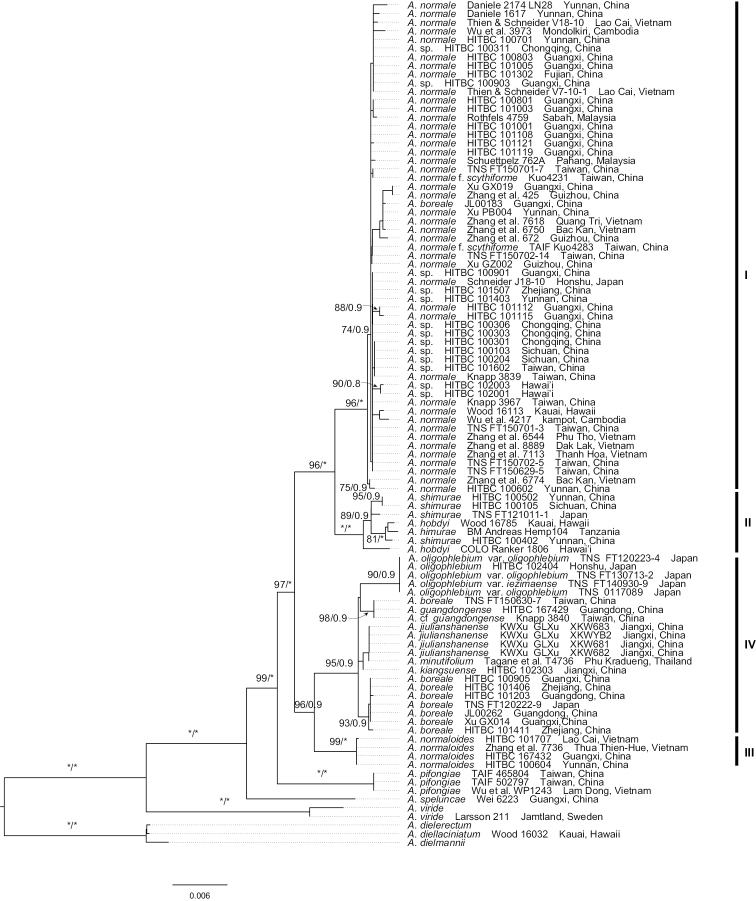
The phylogenetic position of *Aspleniumjiulianshanense* K.W.Xu & G.L.Xu based on seven plastid markers (*atpB*, *rbcL*, *rps4*, *rps4-trnS*, *trnL*, *trnL-F*, and *trnG*). The numbers associated with branches are maximum likelihood bootstrap (MLBS) values followed by Bayesian inference posterior probabilities (PP). “*” indicates MLBS = 100% or PP = 1. Black vertical bars indicate those major clades identified by Chang et al. (2018).

**Figure 4.**
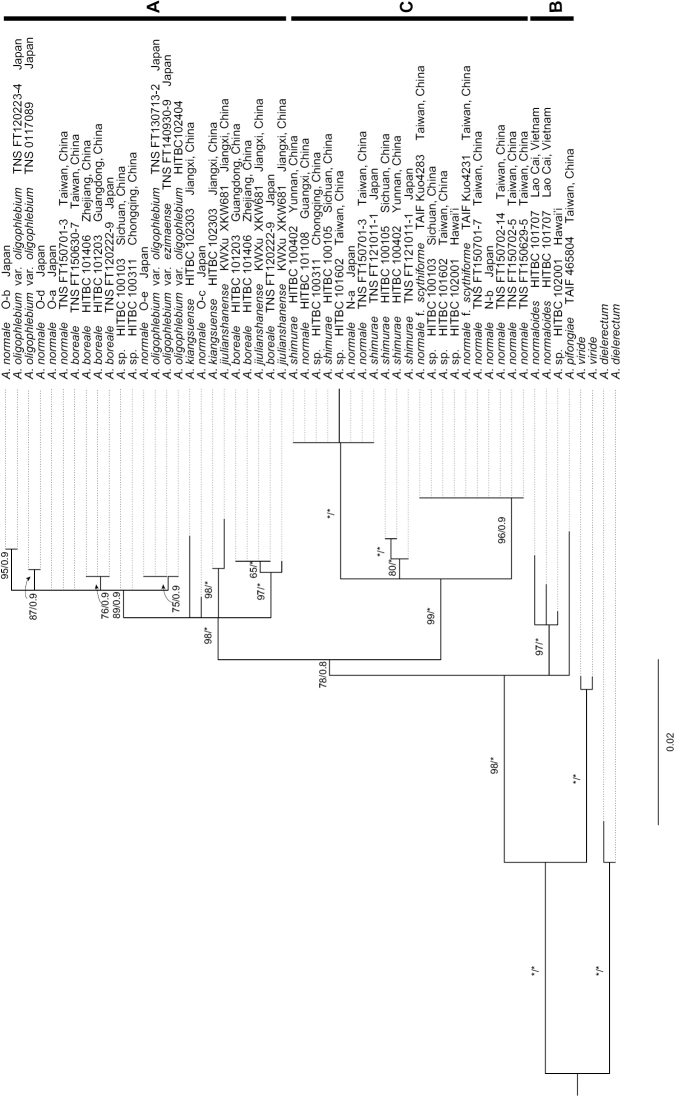
The phylogenetic position of *Aspleniumjiulianshanense* K.W.Xu & G.L.Xu based on the low-copy nuclear gene *pgiC*. The numbers associated with branches are maximum likelihood bootstrap (MLBS) values followed by Bayesian inference posterior probabilities (PP). “*” indicates MLBS = 100% or PP = 1. Black vertical bars indicate those major clades identified by Chang et al. (2018).

In 2020, we collected a peculiar specimen of the *A.normale* complex from the Jiulianshan National Nature Reserve, China. It usually has one gemma near each rachis apex, pinna margins entire to sparsely crenate, and each pinna has (1‒)3‒4(‒6) sori (Figs 1, 2). It could not be identified and assigned to any currently recognized species in the *A.normale* complex based on its gross morphology. Our further phylogenetic analyses, however, revealed that this specimen is closely related to, but distinct from, *A.kiangsuense* (Figs 3, 4). Further examination of scale and spore morphology suggested that this entity represents a distinct species new to science. Therefore, we described and illustrated the new species of the *A.normale* complex and provided a key to all related species in the complex.

## ﻿Materials and methods

### ﻿Fieldwork and morphological study

Under the cooperative project with Jiulianshan National Nature Reserve (#202102220001), several botanical explorations were made to the Jiulianshan National Nature Reserve and extensive field investigations of the new species were conducted from 2020 to 2021 by us. The gross morphology of the new species was photographed and the quantitative characters were measured when conducting field investigations. Herbarium specimens were collected and deposited in the herbaria NF and SYS. Herbarium abbreviations follow Thiers (2020). A systematic examination of online digital images of the *A.normale* complex available on CVH (https://www.cvh.ac.cn/) and GBIF (https://www.gbif.org/) was carried out. Scanning Electron Microscope (SEM) was used to take spore images of the new species. Mature spores obtained from herbarium specimen were mounted on specimen tabs and then coated with platinum in a sputter coater. Observations were conducted using an ESEM-Quanta 200 (FEI, Hillsboro, Oregon, US) with 15 Kv at Nanjing Forestry University, Nanjing, China. ImageJ software (Rasband 1997–2017) was used to measure the morphological data and the SEM micrographs.

### ﻿Cytological study

The young root tips of the new species were pretreated with a mixture of 2mM 8-hydroxyquinoline solution and 0.2% colchicine solution (volume ratio = 1: 1) for 3 h and then fixed in Carnoy’s solution for 5 h. The tips were then macerated in 1 N HCl at 60 °C for 10 minutes and then squashed in 2% aceto-orcein. The chromosomes of the two samples of the new species were counted and photographed using a light microscope (Olympus, Japan).

### ﻿Phylogenetic study

Phylogenetic analyses of the *A.normale* complex were performed to study the interspecific relationships among the new species and other members in the complex based on sequences of seven plastid markers (*atpB*, *rbcL*, *rps4*, *rps4-trnS*, *trnL*, *trnL-F*, and *trnG*; with *trnL* and *trnL-F* combined and *rps4* and *rps4-trnS* combined) and one low-copy nuclear gene, *pgiC*. Total genomic DNA was extracted from silica gel-dried leaves using the modified 2 ×CTAB procedure of Doyle and Doyle (1987). Primers and PCR protocols followed Chang et al. (2018) and Xu et al. (2020). For the nuclear gene *pgiC*, the purified nuclear DNA products were ligated into a pUCm-T Vector (Sangon, Shanghai, China). Ten positive clones for each individual were randomly selected for sequencing. Except for the new species, all other DNA sequences of the *A.normale* complex used in this study were downloaded from NCBI (https://www.ncbi.nlm.nih.gov/) following Chang et al. (2018) and Xu et al. (2020). Voucher information and GenBank accession numbers are provided in Suppl. material 1.

The newly generated sequences were assembled and edited using Sequencher V.4.14 (GeneCodes Corporation, Ann Arbor, Michigan). All sequences of each gene were initially aligned with MAFFT v.7 (Katoh and Standley 2013) and manually adjusted in BioEdit (Hall 1999). The aligned sequences of seven plastid genes were then concatenated using PhyloSuite (Zhang et al. 2020). Independent phylogenetic analyses of the plastid and nuclear datasets were conducted using RAxML (Stamatakis 2006) and MrBayes v. 3.2.7a (Ronquist and Huelsenbeck 2003) on the Cipres web server (Miller et al. 2010), respectively. The maximum likelihood (ML) tree searches were performed using RAxML-HPC2 on XSEDE with 1000 bootstrap replicates. The models of nucleotide substitution for the combined plastid DNA dataset (TIM2+I+G) and the nuclear *pgiC* (F81+I+G) were selected independently under the Akaike Information Criterion (AIC) using jModelTest v. 3.7 (Darriba et al. 2012). The temperature parameter of Bayesian inference (BI) set to 0.2, and other priors set to their default values. Two independent runs, each with four chains (one cold, three heated), were conducted, each beginning with a random tree and sampling one tree every 1000 generations of 10 000 000 generations. Convergence among runs and stationarity was assessed using TRACER v.1.4 (Rambaut and Drummond 2007), and the first 25% was discarded as burnin. The remaining trees were used to calculate a 50% majority-rule consensus topology and posterior probabilities (PP).

## ﻿Results

### ﻿Ploidy analyses

**Figure 5.**
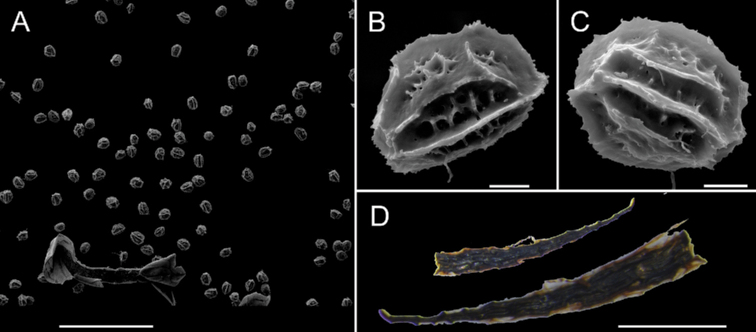
*Aspleniumjiulianshanense* K.W.Xu & G.L.Xu **A–C** spore morphology of the new species **D** rhizome scale. Scale bars: 300 μm (**A**); 10 μm (**B, C**); 1 mm (**D**).

**Figure 6.**
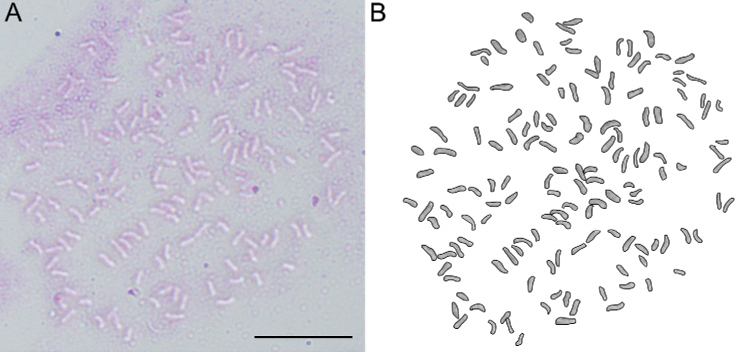
Chromosomes of *Aspleniumjiulianshanense* K.W.Xu & G.L.Xu in mitotic root-tip cells, 2n = 144. Scale bars: 20 μm.

The spores of the new species *Aspleniumjiulianshanense* were well-developed (Fig. 5A). We counted the spore number per sporangium under a light microscope and found that each sporangium contains 64 spores. Therefore, we assumed that it is sexually reproducing. Spore size is considered a good indicator of ploidy level when compared with close relatives in the *A.normale* complex (Chang et al. 2013, 2018). We measured the spore size of the new species (37‒43 µm, Fig. 5) and calculated the mean spore size (39.8 µm) based on 20 mature spores using ImageJ. It has a larger spore size than most members of the complex, indicating polyploidy (Fujiwara et al. 2017; Chang et al. 2018). In addition, our cytological study confirmed that the chromosome number of the new species is 2n = 144 (Fig. 6), which indicates that it is a tetraploid species.

### ﻿Plastid gene phylogenetic analyses

The 98 aligned plastid gene sequences are 5,583 bp in length, with 382 parsimony informative sites in total. The tree topologies by the ML and BI analyses were generally concordant when using the concatenated plastid dataset. The major clades of the phylogeny reconstructed in this study were also congruent with those of previous studies (Chang et al. 2018, 2020; Xu et al. 2020). Owing to only one gene region of *rbcL* with 525 bp being available from NCBI for *A.minutifolium* Kanem. & Tagane, four collections of the new species and one collection of *A.minutifolium* together formed a well-supported clade nested within clade III, and this well-supported clade was resolved as sister to *A.kiangsuense* (Fig. 3).

### ﻿Low-copy nuclear gene phylogenetic analyses

The nuclear dataset included 54 aligned sequences in total. The total alignment was 851 bp in length, with 98 parsimony informative sites. All the three *pgiC* alleles of the new species were nested within clade A. Within clade A, one allele of the new species was well resolved as sister to *A.kiangsuense*, while the other two alleles were closely related to some alleles of *A.boreale* (Ohwi ex Sa. Kurata) Nakaike (Fig. 4).

### ﻿Taxonomic treatment

#### ﻿ Asplenium jiulianshanense

Taxon classificationPlantaePolypodialesAspleniaceae

K.W.Xu & G.L.Xu sp. nov.

C3E1B79C-C861-519E-86F9-714DA33A46C3

urn:lsid:ipni.org:names:77299335-1

Figs 1 , 2 , 5

##### Diagnosis.

*Aspleniumjiulianshanense* somewhat resembles *A.kiangsuense* by its small size, rachises adaxially without a deep furrow, pinnae elliptic to trapeziform-oblong, sori 3–4 per pinna in the middle part of frond. However, the former has rachises with only one gemma near apex, pinnae (15‒)20‒35 pairs, pinna margins entire to sparsely crenate, exospore length 37‒43 μm, while the latter has rachises without gemmae near the apex, pinnae 8‒20(‒22) pairs, pinna margins entire to sinuate, exospore length 31‒36 μm.

##### Type.

China. Jiangxi province, Ganzhou City, Jiulianshan National Nature Reserve, 256 m, 24°56'2.04"N, 114°49'23.44"E, 8 Jun 2021, *Guo-Liang Xu & Ke-Wang Xu XKW681* (holotype, NF!; isotype, SYS!).

##### Description.

Plants 8‒15 cm tall. Rhizome erect, short, apex densely scaly; scales narrowly triangular to linear-subulate, purplish-black, 2‒3 × 0.4‒0.6 mm. Fronds caespitose; stipe castaneous-brown to purplish-black, shiny, terete, 1‒3 cm, glabrous; lamina linear, 8‒12 × ca. 1.2 cm, apex acute, 1-pinnate; pinnae (15‒)20‒35 pairs, lower ones subopposite, hardly reduced, middle pinnae spreading horizontally or slightly reflexed, elliptic to trapeziform-oblong, 3‒6 × 3‒5 mm, base asymmetrical, acroscopic side truncate and close to rachis, basiscopic side narrowly cuneate, shortly stalked to subsessile, margin entire to sparsely crenate, apex obtuse. Venation anadromously pinnate or with first basiscopic vein lacking, costa with 2 or 3 acroscopic veins, obscure, veins simple or 1-forked. Fronds papery, grayish-green when dry; rachis castaneous-brown to purplish-black, shiny, glabrous, semiterete, and adaxially flat or with 2 slightly raised lateral ridges, often gemmiferous near the apex. Sori (1‒)3‒4(‒6) per pinna, linear-elliptic, ca. 1 mm in length, median on subtending vein; indusia grayish-green, elliptic, membranous, entire, and opening toward costa. Spores with lophate perispore, average exospore length 37‒43 µm.

##### Etymology.

Based on the Chinese pinyin, Jiulianshan, the name of the National Nature Reserve in southern Jiangxi, China, referring to the type locality of the species.

##### Vernacular name.

九连山铁角蕨 (jiǔ-lián-shān tiě-jiǎo-jué).

##### Geographical distribution and habit.

*Aspleniumjiulianshanense* is known only from a single locality in Mount Jiulianshan, Jiangxi, China, where there have been multiple collections. It was observed to grow on cliff rocks under shrubs at an elevation of ca. 200 m in subtropical evergreen broad-leaved forest.

##### Conservation.

We provisionally assess *A.jiulianshanense* as Endangered based on criterion D of IUCN (2012). Only one population of the new species was found at type locality with no more than 250 mature individuals.

##### Cytology.

The chromosome number of *A.jiulianshanense* is 2n = 144 (Fig. 6). The chromosome number of *A.jiulianshanense* indicates that the new species is a tetraploid species.

##### Additional specimens examined.

China. Jiangxi province, Ganzhou City, Jiulianshan National Nature Reserve, 256 m, 24°56'2.04"N, 114°49'23.44"E, 10 Jul 2021, *Guo-Liang Xu & Ke-Wang Xu XKW685* (NF!); the same collection information, *Guo-Liang Xu & Ke-Wang Xu XKW686* (NF!).

### ﻿A key to *Aspleniumjiulianshanense* and its closely related taxa revised from Chang et al. (2018)

**Table:** 

1	Pinna dissected	**2**
–	Pinna subentire	**3**
2	Pinna falcate; bud not elongated to whip-shape	**A.normalef.scythiforme**
–	Pinna linear; bud elongated to whip-shape	***A.oligophlebium***
3	Fronds with no buds on the rachis	**4**
–	Fronds with buds on the rachis	**8**
4	Rachis adaxially without a deep furrow	**5**
–	Rachis adaxially with a deep furrow	**6**
5	Laminae 0.7 cm in width; rachises wingless; sori usually arranged in a row	***A.minutifolium***
–	Laminae ca.1 cm in width; rachises with slightly raised lateral wings; sori arranged oppositely	***A.kiangsuense***
6	Sori less than 5 and mostly only on, and parallel to, the basiscopic side of pinnae	***A.pifongiae***
–	Sori normally more than 5 and on both basiscopic side and acrosopic sides of pinnae	**7**
7	Mean spore size 27‒32 µm	***A.guangdongense***
–	Mean spore size 34‒37 µm	***A.boreale***
8	Frond buds at both the distal end and middle part of the rachis	**9**
–	Frond buds only at the distal end of rachis	**11**
9	Mean spore size 27‒32 µm	***A.pseudonormale***
–	Mean spore size 34‒37 µm	**10**
10	Pinna apices obtuse; acroscopic margin of pinnae deeply undulate; endemic to Hawaii	***A.hobdyi***
–	Pinna apices acute; acroscopic margin of pinnae slightly undulate; endemic to Japan	***A.shimurae***
11	Plants less than 15 cm tall, sori 2‒4 per pinna	***A.jiulianshanense***
–	Plants more than 15 cm tall, sori more than 4 per pinna	**12**
12	Spores with highly perforate perispore; sori 10‒14 per pinna; diploids only	***A.normaloides***
–	Spores without perforate perispore or only slightly perforate; sori usually less than 10 per pinna; diploids or tetraploids	**13**
13	Pinnae acroscopically auriculate to hastate, margin deeply serrate; tetraploids only	***A.serratipinnae***
–	Pinnae acroscopically truncate, margin subentire to crenate; diploids and tetraploids	***A.normale***

## ﻿Discussion

### ﻿Taxonomy of the *Aspleniumnormale* complex

The *A.normale* complex is widely distributed in south and southeast Asia, tropical east Africa, and tropical Pacific islands (Chang et al. 2013; Lin and Viane 2013). However, the species delimitation and taxonomic treatment within the complex have been controversial until an integrative taxonomic approach was employed (Wu 1999; Chang et al. 2013, 2018; Lin and Viane 2013). The treatment of one species *A.normale* with some varieties was generally accepted by former studies (Kurata 1963; Ito 1972; Wu 1999). Recent integrative taxonomic studies using cytological, morphological, and DNA sequence data revealed that recurrent reticulation events occurred among members of this species complex (Chang et al. 2013, 2018). The status of an increasing number of taxa within the species complex has been confirmed using integrative taxonomy in recent studies (Chang et al. 2013, 2018, 2020; Fujiwara et al. 2017, 2020).

Chang et al. (2018) recognized seven species (*A.boreale*, *A.guangdongense*, *A.kiangsuense*, *A.normaloides*, *A.normale*, *A.pifongiae*, *A.pseudonormale*) of the *A.normale* complex in China. Morphologically, the new species *A.jiulianshanense* collected from the Jiulianshan National Nature Reserve is most similar to *A.kiangsuense* in having plants less than 15 cm tall, rachises adaxially without a deep furrow, pinnae elliptic to trapeziform-oblong, and 3–4 sori per pinna in the middle part of frond (Fig. 1). However, the new species can be easily distinguished from *A.kiangsuense* by fronds with only one gemma at the distal end of rachis, 8‒20(‒22) pinnae pairs, pinna margins entire to sinuate, exospore length 37‒43 µm (Fig. 1). We also checked the synonyms (e.g. *A.gulingense* Ching & S. H. Wu, *A.hangzhouense* Ching & C. F. Zhang, *A.parviusculum* Ching) related to *A.kiangsuense*. All of them lack gemmae at the distal end of rachis and those pinnae in the lower part of the frond are spreading and divaricate, while *A.jiulianshanense* usually has only one gemma at the distal end of rachis and pinnae in the lower part of the frond are spreading horizontally or reflexed. Within taxa bearing gemmae on the rachis, the gemmae of the new species only occur at the distal end of rachis, which is also different from *A.pseudonormale*. The new species can also be distinguished from *A.normaloides* and *A.normale* by having plants less than 15 cm tall (vs. more than 20 cm tall in *A.normaloides* and more than 15 cm tall in *A.normale*) and 3–4 sori per pinna in the middle part of the frond (vs. 10–14 sori per pinna in *A.normaloides* and usually more than 4 sori per pinna in *A.normale*). In addition, Kanemitsu et al. (2017) described a new species *A.minutifolium* of the *A.normale* complex from Thailand, which is also similar to *A.jiulianshanense* in morphology. However, *A.jiulianshanense* is distinct from *A.minutifolium* by usually having one gemma at the distal end of each rachis (vs. with no buds on the rachis in *A.minutifolium*), pinna margins entire to sparsely crenate (vs. pinna margins entire or slightly undulate in *A.minutifolium*), and sori arranged oppositely (vs. usually arranged in a row in *A.minutifolium*).

### ﻿Reticulate evolution in the *A.normale* complex and origin of *A.jiulianshanense*

The reticulate relationships of the *A.normale* complex have been well investigated using evidence from molecular, cytological, and morphological data (Chang et al. 2013, 2018, 2020; Fujiwara et al. 2017). Both diploids and tetraploids were confirmed to exist in the complex and multiple tetraploid species were revealed to originate via autopolyploidy and allopolyploidy (Chang et al. 2013; Fujiwara et al. 2017). For example, the allotetraploid species *A.serratipinnae* T.Fujiw. & Watano originated from their diploid parents of *A.normale* and *A.oligophlebium* (Fujiwara et al. 2017, 2020), and autotetraploid species *A.kiangsuense* and *A.boreale* Nakaike were revealed by the evidence that their two *pgiC* copies nested within the same group (Chang et al. 2013).

For the case of the new species *A.jiulianshanense*, both the spore size and the chromosome number indicate that it was a tetraploid species. The count of 64 spores per sporangium suggests that the new species has sexual reproduction. Phylogenetically, though only one gene region *rbcL* with 525 bp was available for *A.minutifolium* and all the 525 sites were identical to those of *A.jiulianshanense* and *A.kiangsuense*, *A.jiulianshanense* and *A.minutifolium* together were well resolved as a distinct clade sister to *A.kiangsuense* based on the phylogeny reconstructed using seven maternally inherited plastid genes (Fig. 3). As for the phylogeny reconstructed using the low-copy nuclear *pgiC* gene, one allele was resolved as sister to the autotetraploid species *A.kiangsuense* and the other two alleles were nested together with the autotetraploid *A.boreale* (Fig. 4). The plastid and nuclear phylogenies together suggest that the present new species is derived from hybridization between autotetraploids *A.kiangsuense* and *A.boreale*. In any case, the tetraploid taxa in the *A.normale* complex still need more study. More collections of additional species, including molecular data and chromosome counts, are still needed to analyze their phylogenetic position and better understand their pathways of reticulate evolution.

## Supplementary Material

XML Treatment for Asplenium jiulianshanense

## Supplementary materials

﻿Supplementary material 1 Table S1 Data type voucher information (excel file) Explanation note List of voucher specimens and Genbank accession numbers used in phylogenetic analyses. https://binary.pensoft.net/file/693691Copyright notice: This dataset is made available under the Open Database License (http://opendatacommons.org/licenses/odbl/1.0/). The Open Database License (ODbL) is a license agreement intended to allow users to freely share, modify, and use this Dataset while maintaining this same freedom for others, provided that the original source and author(s) are credited.Chen-Xue Lin, Guo-Liang Xu, Zhi-Fang Jin, Wen-Bo Liao, Ke-Wang Xu
